# Effect of brachytic2 mutation in corn silage on performance, apparent total-tract digestibility, and greenhouse gas emissions of lactating Holsteins fed 2 concentrations of dietary neutral detergent fiber

**DOI:** 10.3168/jdsc.2026-1033

**Published:** 2026-06-11

**Authors:** E. Sarmikasoglou, R.E. Barber, T. Dietz, D. Hammer, M.J. VandeHaar

**Affiliations:** 1 Department of Animal Science, Michigan State University, East Lansing, MI 48824; 2 Bayer Crop Science, Chesterfield, MO 63141

## Abstract

Corn hybrids carrying the *brachytic2* (*br2*) mutation have reduced plant height and improved plant standability. The objective of this study was to evaluate the effect of feeding corn silage (CS) from *br2* corn with CS from conventional tall corn on feed intake, productivity, total-tract nutrient digestibility, and greenhouse gas emissions of Holstein cows fed diets with high or low dietary NDF. Twenty primiparous and 20 multiparous Holstein cows (76 ± 31 DIM, 46 ± 9.0 kg milk/d, 658 ± 83 kg BW) were enrolled in a randomized complete block design with a 14-d pretreatment period in which all cows were fed a common diet and a 42-d period in which cows were fed 1 of 4 diets. Experimental diets contained *br2* or tall CS at 2 dietary NDF contents (25% and 30%). Low-NDF diets had 17% CP, 32% starch, and 17% forage NDF (forNDF); high-NDF diets had 18% CP, 23% starch, and 24% forNDF. Data were analyzed using PROC GLIMMIX of SAS 9.4. Contrasts were used to determine the effect of CS hybrid, the effect of NDF content, and their respective interaction. Cows fed *br2* CS had greater DMI (27.5 vs. 26.5 kg), ECM (44.6 vs. 42.9 kg/d), and protein yield (1.38 vs. 1.33 kg/d) than did cows fed tall CS. Cows fed low-NDF diets had greater DMI (25.9 vs. 28.2 kg), yields of milk (42.6 vs. 45.5 kg/d), and protein (1.28 vs. 1.42 kg/d), but lower ECM/DMI (1.73 vs. 1.64) than cows fed high-NDF diets. The apparent total-tract digestibilities of NDF and CP were greater for diets with *br2* than tall CS, and high-NDF diets had greater total-tract digestibilities of NDF, starch, and CP than low-NDF diets. Lastly, CH_4_ output (520 vs. 460 g/d), CH_4_/DMI (20.1 vs. 17.0 g/kg), and CH_4_/ECM (11.5 vs. 10.1 g/kg) were greater for cows fed high NDF than low NDF but not altered by corn hybrid. We observed no interactions of hybrid × fiber content, except for DMI, where the increase in DMI from feeding *br2* compared with tall CS was greater in the high-silage, high-NDF diets. In conclusion, *br2* CS increased productivity of lactating cows compared with CS from a tall hybrid of similar NDF and starch content.

## Highlights

• The *br2* short-stature corn silage increased DMI and ECM compared with tall corn silage.
• The NDF digestibility was greater in cows fed br2 than in those fed tall corn silage.
• Production, yield, and intensity of CH4 were similar between cows fed br2 and the tall corn silage.

## Graphical Abstract

**Figure:**
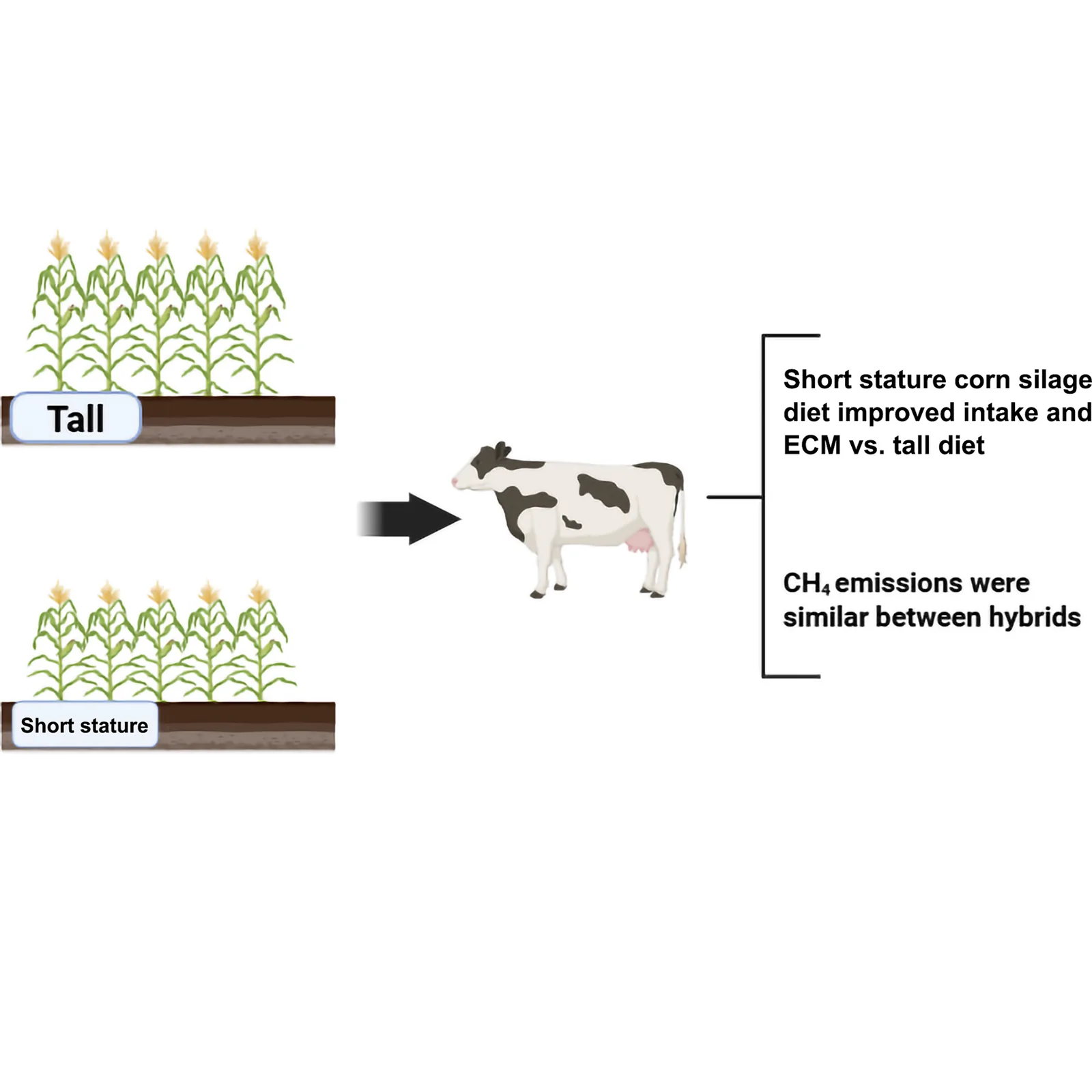
**Summary:** Corn hybrids that carry the *brachytic2* (*br2*) mutation have reduced plant height and improved plant stability relative to conventional tall hybrids. We fed Holstein cows diets with high or low neutral detergent fiber (NDF) that contained corn silage (CS) from a short-stature hybrid with the *br2* mutation or CS from a conventional tall hybrid and compared their effects on intake, productivity, total-tract nutrient digestibility, and greenhouse gas emissions. Cows fed low-NDF diets had greater dry matter intake (DMI) and yields of milk, but lower feed efficiency than cows fed high-NDF diets. Cows fed *br2* CS had greater DMI and produced more energy-corrected milk (ECM) than cows fed tall CS, and these *br2* effects were greatest in the high-NDF diet. The CH4 output, CH4/DMI, and CH4/ECM were lower for cows fed low-NDF diets and were not altered by corn hybrid. Overall, *br2* CS improved the intake and performance of lactating cows compared with CS from a tall hybrid containing similar NDF. Created with BioRender.com.

High-quality forages are essential to enable modern dairy cows to reach their genetic potential for milk production. High-quality forages have effective fiber to promote gut health, and the fiber has high digestibility so that it clears quickly from the rumen to enable greater feed intake and provide more energy for milk synthesis (Oba and Allen, 2000a,b). Corn silage (**CS**) is commonly used as a forage source in lactating dairy cow diets because it has effective and digestible fiber, is approximately one-third starch, and increases milk production compared with most other forages (Oba and Allen, 1999; Ferraretto and Shaver, 2012). Corn silage from hybrids with lower lignin content, such as *bm3* brown midrib hybrids, can be more digestible than conventional CS and increase DMI and milk production (Oba and Allen, 2000b; Ebling and Kung, 2004). However, *bm3* hybrids have disadvantages; most notably, they have lower DM yields, lower starch content, and decreased standability compared with conventional hybrids (Sattler et al., 2010; Wallau et al., 2022).

Short-stature corn hybrids carrying the natural mutation *brachytic2* (***br2***) exhibit shorter internodes, more erect leaves, and reduced plant height compared with conventional tall hybrids (Pilu et al., 2007; Barten et al., 2022). In the field, these characteristics of *br2* corn hybrids result in greater plant standability and decreased losses in productivity during windstorms (Barten et al., 2022). Byers and coworkers (1965) first examined the feeding potential of CS from dwarf corn to dairy cattle and found no effects on DMI, FCM, or BW change; whether those dwarf hybrids were carrying the *br2* mutation is not known. Previous reports that examined the feeding potential of silage from *br2* corn hybrids in lactating diets found improvements in milk and protein yield compared with a tall corn hybrid (Sarmikasoglou et al., 2025; Catellani et al., 2026), whereas others reported no effects on DM yield parameters (Mastroeni et al., 2026). The objective of this study was to evaluate the effect of feeding CS from *br2* corn, compared with conventional tall corn, on feed intake, productivity, total-tract nutrient digestibility, and greenhouse gas emissions of Holstein cows fed diets with 2 concentrations of dietary NDF.

All experimental procedures were approved by the Michigan State University Institutional Animal Care and Use Committee (PROTO202300255). A conventional mid-height corn hybrid (tall; DKC59-07RIB, DEKALB, Bayer Crop Science, Leverkusen, Germany) and a *br2* short-stature hybrid (RV6210TVXZ, Bayer Crop Science) were each planted at 86,500 seeds/ha in adjacent fields plots of 2.19 ha each at Michigan State University (East Lansing, MI), on May 15, 2023, and grown under the same tillage, fertilizer application, and weed control practices. The relative maturities of tall and *br2* hybrids were 109 and 112 d, respectively. The selection of this *br2* hybrid was based on the lactation performance from a previous study in which we compared 3 novel short-stature corn hybrids and the tall hybrid (Sarmikasoglou et al., 2025). Harvesting and ensiling procedures are described in detail in that study (Sarmikasoglou et al., 2025), which was conducted with same silages several months earlier. Briefly, corn forages were chopped at 34% DM, ensiled in separate silage bags and allowed to ferment for 13 mo for this study. Yields of DM harvested were 19 and 21 t/ha for the tall and *br2* hybrids, respectively. Based on analyses of samples collected at 6-m intervals on the sides of each bag by Cumberland Valley Analytical Services (Waynesboro, PA), the in vitro neutral detergent fiber digestibility (%NDF) after 30 h of incubation of tall and *br2* CS was 53% ± 0.8% and 60% ± 1.3%, respectively. The nutrient composition of the CS is presented in footnotes of Table 1.

**Table 1:** Ingredient and nutrient composition of experimental diets

Item	Low NDF		High NDF	
	*br2*	Tall	*br2*	Tall
% DM				
*br2* corn silage^1^	35.5	—	55.7	—
Tall corn silage^2^	—	36.0	—	56.5
Alfalfa haylage	14.0	14.0	14.0	14.0
Corn, dry ground	23.0	22.0	1.6	—
Soybean meal	10.0	10.5	11.2	12.0
Cottonseed	7.00	7.00	7.00	7.00
Protein premix^3^	8.00	8.00	8.00	8.00
Mineral and vitamin premix^4^	2.50	2.50	2.50	2.50
Nutrient composition				
aNDFom (% of DM)	24.7	25.0	29.9	30.3
forNDF (% of DM)	16.8	17.2	23.9	24.5
NEL^5^ (Mcal/kg)	1.67	1.67	1.63	1.63
CP (% of DM)	17.7	17.7	18.1	18.2
Starch (% of DM)	31.6	31.6	22.7	22.7
Fatty acids (% of DM)	3.57	3.56	3.33	3.31

1 *br2* corn silage (33.0% ± 0.43% DM). Nutrient composition (% DM) was 36.5% ± 0.85% NDF, 21.9% ± 0.29% ADF, 2.32% ± 0.09% lignin, 8.13% ± 0.19% CP, and 35.2% ± 0.49% starch.

2 Tall corn silage (34.5% ± 0.71% DM). Nutrient composition (% DM) was 35.9% ± 0.66% NDF, 22.0% ± 0.86% ADF, 2.74% ± 0.14% lignin, 7.10% ± 0.22% CP, and 40.4% ± 1.62% starch.

3 Protein premix contained (% as fed) 40.82% Amino Plus (Ag Processing Inc.), 17.69% corn grain, 14.64% sodium sesquinate, 11.97% Spectrum AgriBlue (Perdue AgriBusiness), 11.9% calcium carbonate, 2.45% urea, and 0.53% Smartamine M (Adisseo).

4 Mineral and vitamin premix contained (% as fed) 20.45% calcium carbonate, 14.72% corn grain, 14.15% salt, 12.49% calcium phosphate, 9.15% magnesium oxide, 4.71% magnesium sulfate, 4.47% sodium sesquicarbonate, 0.92% tallow, and trace minerals and vitamins to meet NASEM (2021) requirements.

5 Based on NASEM (2021), assuming no difference in the digestibility of silages.

The study was conducted from September to November of 2024 at the Michigan State University Dairy Cattle Teaching and Research Center (East Lansing, MI). Twenty primiparous, and 20 multiparous Holstein cows (76 ± 31 DIM, 46 ± 9 kg milk/d, 658 ± 83 kg BW) were enrolled in a randomized complete block design. One cow was removed from the study due to clinical mastitis. Cows were assigned to blocks based on DIM, parity, and milk yield per unit of metabolic BW. A total of 10 blocks of 4 cows were established. Each cow within a block was randomly assigned to 1 of the 4 treatments. Cows were housed in individual tiestalls and milked thrice daily. Water was available ad libitum. Feed was offered once daily at 0800 h at 115% of expected intake.

Cows were fed a common diet for a 14-d covariate period and then experimental diets for 42 d. Experimental diets contained either *br2* or conventional tall CS, alfalfa silage, soybean meal, ground corn, whole cottonseed, a protein supplement premix, and a premix of minerals and vitamins (Table 1). Low-NDF diets contained ∼36% CS and 22% ground corn grain and had 17% CP, 32% starch, and 17% forage NDF (**forNDF**), whereas high-NDF diets contained ∼56% CS and ∼0% to 1.6% ground corn grain and had 18% CP, 23% starch, and 24% forNDF. Diets were formulated to contain the same NDF, forNDF, CP, starch, and fat concentrations within NDF level and fed as TMR. Minerals and vitamins were formulated according to NASEM (2021).

Milk yield was recorded daily, and milk samples were collected weekly at every milking for 2 consecutive days. Samples were stored with preservative (Bronolab W-II liquid, Advanced Instruments) at 4°C until analysis. Individual milk samples were analyzed by CentralStar Cooperative Inc. for fat, true protein, lactose, MUN, and SCC concentrations by mid-infrared spectroscopy (AOAC, 1990). Milk yield and component concentrations for each milking were summed for a daily total and to calculate the ECM and milk component yields. The ECM was calculated as follows:

$$ECM=\frac{\left(NEL_{NASEM,2021}\times kg\;of\;milk\right)}{0.7},$$

where milk energy output NEL (Mcal/kg) for each cow was estimated by the following equation (NASEM, 2021, Equation 3-14b):

$$\begin{array}{l} NEL\;\left(Mcal/kg\right)=9.29\times \;\frac{kg\;fat}{kg\;milk}+5.85\times \frac{kg\;true\;protein}{kg\;milk}\\ +3.95\times \frac{kg\;lactose}{kg\;milk}. \end{array}$$

Cows were weighed 3 consecutive days on wk −1, 3, and 6 immediately following afternoon milkings. Body condition score for each cow was measured as the average score of 3 trained investigators and was recorded on a 5-point scale on wk −1, 3, and 6, with a score of 1 reflecting cows that are emaciated and a score of 5 reflecting cows that are obese.

Samples of all diet ingredients and TMR (∼0.5 kg) were collected once weekly for 6 weeks and stored at −20°C until composited and dried; these values were used for calculating final diet composition (Table 1). Apparent total-tract digestibilities were determined for each cow over 3 consecutive days on wk 6. Samples of feed ingredients (∼0.5 kg) and orts (12.5% of remaining feed) for individual cows were collected daily and composited. Feces (∼400 g) were collected every 9 h over the 3 d, resulting in 8 samples/cow representing every 3 h of a day. Feces were stored at −20°C until dried and composited on an equal DM basis for each cow. Diet ingredients, orts, and fecal samples were dried at 55°C for 72 h in a forced-air oven to determine DM. Dried samples were ground with a Wiley mill (5-mm screen; Arthur H. Thomas). Samples of diet ingredients and feces were analyzed by Cumberland Valley Analytical Services (Waynesboro, PA) for CP (AOAC International, 2000), starch (Hall, 2009), and ash (method 142 942.05; AOAC International, 2000) modified to ash a 1.50-g sample for 4 h. The NDF and undegradable NDF (**uNDF**) were determined according to Mertens (2002) and Goering and Van Soest (1970), respectively. The uNDF, estimated as NDF residue after 240 h of in vitro fermentation, was used as an internal marker to predict fecal output (Cochran et al., 1986). Apparent total-tract digestibilities of nutrients were calculated as in Daniel et al. (2020) with the assumption that uNDF intake from TMR is equal to uNDF fecal output.

Spot sampling measurements of CO_2_, O_2_, CH_4_, and H_2_ were obtained from individual cows by using 2 GreenFeed units (**GF**; C-Lock Inc., Rapid City, SD) in a naturally ventilated tiestall barn. Greenhouse gas measurements were collected at wk 3 and 6 every 9 h over the 3 d, resulting in 8 samples/cow representing every 3 h over a day. The GF was set to dispense 8 drops (35 g per drop) of pellets per sampling at 30-s intervals to ensure 5 min of continuous measurement. Dehydrated alfalfa pellets (17% CP, 33% crude fiber, DM basis) were dispensed from the GF as bait to entice cows to use the head chamber during measurement.

Normality of residuals and homogeneity of variance were examined for each continuous dependent variable. Means for response variables were used as repeated measures in a model. The most appropriate covariance structure was selected for each model based on the smallest corrected Akaike information criterion. All statistical analyses were performed using the GLIMMIX procedure of SAS 9.4 (SAS Institute Inc., Cary, NC) according to the following model:

*Y_ijklmn_* = Cov + *μ* + *H_i_* + *F_j_* + *W_k_* + *P_l_* + *B_m_*(*P_l_*) + *C_n_*(*B_m_* × *H_i_*) + *C_n_*(*B_m_* × *F_j_*) + *H_i_* × *F_j_* + *W_k_* × *H_i_* + *W_k_* × *F_j_* + *P_l_* × *H_i_* + *P_l_* × *F_j_* + *P_l_* × *W_k_* + *H_i_* × *F_j_* × *W_k_* + *P_l_* × *H_i_* × *F_j_* + *P_l_* × *W_k_* × *F_j_* + *P_l_* × *W_k_* × *H_i_* + *e_ijklmn_*,

where *Y_ijklmn_* is the observation *ijklmn*; Cov is the true covariate; *µ* is the overall mean; *H_i_* is the fixed effect of hybrid (*i* = *br2* vs. tall); *F_j_* is the fixed effect of fiber level (*j* = low vs. high); *W_k_* is the fixed effect of week (*k* = 1 to 6); *P_l_* is the fixed effect of parity (*l* = primiparous vs. multiparous); *B_m_*(*P_l_*) is the random effect of block nested with parity; *C_n_*(*B_m_* × *H_i_*) is the random effect of cow nested with block and hybrid (*m* = 1–10, *n* = 1–4); *C_n_*(*B_m_* × *F_j_*) is the random effect of cow nested with block and fiber level; *H_i_* × *F_j_* is the interaction between hybrid and fiber level; *W_k_* × *H_i_* is the interaction between week and hybrid; *W_k_* × *F_j_* is the interaction between week and fiber level; *P_l_* × *H_i_* is the interaction between parity and hybrid; *P_l_* × *F_j_* is the interaction between parity and fiber level; *P_l_* × *W_k_* is the interaction between parity and week; *H_i_* × *F_j_* × *W_k_* is the interaction between hybrid, fiber, and week; *P_l_* × *H_i_* × *F_j_* is the interaction between parity, hybrid, and fiber level; *P_l_* × *W_k_* × *F_j_* is the interaction between parity, week, and fiber level; *P_l_* × *W_k_* × *H_i_* is the interaction between parity, week, and hybrid; and *e_ijklmn_* is the random residual. Greenhouse gases, CH_4_/DMI, and CH_4_/ECM were analyzed using the same model but from data collected on wk 3 and 6 and excluding the covariate variable. Body weight change and BCS change were analyzed using the same model but from data collected during the covariate period (wk −1) and wk 6. Digestibility measurements were analyzed using the same model but excluding week, all the 2-way and 3-way interactions between week and the other main effects, and the covariate variable. Main effects were declared significant at *P* ≤ 0.05, and tendencies at *P* ≤ 0.10. Interactions were declared significant at *P* ≤ 0.10; interactions with parity and with week were not significant, and only the interaction of dietary NDF content and corn silage hybrid is reported.

On average, cows consumed 27 kg of TMR daily and produced 44 kg of milk daily with 3.6% fat, 3.1% true protein, and 4.8% lactose (Table 2). The interaction of NDF level and hybrid also was significant; in high-NDF diets, cows fed *br2* CS consumed more than cows fed tall CS but in low-NDF diets, intake was similar (*P_H_*_×_*_F_* = 0.08). Moreover, cows fed the *br2* CS produced more ECM (*P_Hybrid_* = 0.05) and protein (*P_Hybrid_* = 0.01) than cows fed tall CS, independent of the NDF level. Cows fed low-NDF diets produced more milk, protein, and lactose and had milk with greater MUN and protein percentage and lower fat percentage (all *P_NDF_* ≤ 0.01) than cows fed high-NDF diets. Moreover, cows fed the low-NDF diets produced less ECM per unit of DMI compared with cows fed the high-NDF diets (*P_NDF_* ≤ 0.01). Captured energy/feed energy in cows fed the diets with tall CS was greater in the high-NDF diet (*P_H_*_×_*_F_* = 0.08). The BW (*P_NDF_* = 0.02) and BW change (*P_NDF_* = 0.01) were lower for cows fed high-NDF diets than cows fed low-NDF diets, regardless of corn hybrid.

**Table 2:** Effects of *brachytic2* (*br2*) mutation of corn and control corn silage at 2 dietary NDF contents on milk production and BW

Variable	Low NDF		High NDF		SEM	*P*-value^1^		
	*br2*	Tall	*br2*	Tall		Hybrid	NDF	H × F
DMI (kg)	28.2	28.1	26.8	25.0	0.61	0.04	<0.01	0.08
Milk (kg/d)	46.2	45.5	43.8	42.1	1.34	0.15	<0.01	0.54
ECM^2^ (kg/d)	45.2	43.3	44.1	42.5	1.18	0.05	0.29	0.84
Fat (%)	3.46	3.37	3.79	3.82	0.10	0.77	<0.01	0.47
Fat (kg/d)	1.58	1.49	1.62	1.57	0.05	0.11	0.17	0.63
Protein (%)	3.13	3.10	3.07	3.01	0.03	0.06	<0.01	0.51
Protein (kg/d)	1.44	1.39	1.33	1.27	0.04	0.01	<0.01	0.71
Lactose (%)	4.88	4.90	4.89	4.86	0.01	0.76	0.26	0.09
Lactose (kg/d)	2.25	2.21	2.14	2.02	0.07	0.10	<0.01	0.41
MUN (mg/dL)	14.3	14.6	17.0	18.7	0.34	0.01	<0.01	0.08
SCC^3^ (×1,000 cells/mL)	12.8	8.87	6.96	8.5	0.89	0.70	0.15	0.19
ECM/DMI	1.67	1.60	1.71	1.75	0.03	0.70	0.01	0.19
Captured energy/feed energy intake^4^	0.30	0.28	0.32	0.31	0.03	0.15	0.10	0.70
BW (kg)	681	677	670	672	3.48	0.66	0.02	0.33
BW change (kg)	0.54	0.42	0.27	0.33	0.07	0.67	0.01	0.17
BCS	3.46	3.42	3.39	3.31	0.07	0.34	0.20	0.71
BCS change (U/42 d)	0.003	0.001	0.003	−0.002	0.002	0.24	0.07	0.42

1 Hybrid = effect of corn silage hybrid; NDF = effect of dietary NDF content; H × F = interaction of dietary NDF content and corn silage hybrid.

2 ECM = (NEL_Equation 3–14b, NASEM, 2021_ × kg milk)/0.7 (Equation 3-14b in NASEM, 2021).

3 Due to nonnormality, SCC was log-transformed for analysis. Means were back-transformed for reporting. The SEM were calculated by using the delta method for SEM estimations.

4 Captured energy/feed energy intake = (milk NEL + body energy change)/DMI × gross energy of feed (NASEM, 2021).

Apparent total-tract digestibilities of NDF (*P_Hybrid_* < 0.01) and CP (*P_Hybrid_* < 0.01) were greater in cows fed *br2* CS than those fed tall silage, and the effect of NDF level on NDF (*P_NDF_* < 0.01), starch (*P_NDF_* < 0.01), and CP (*P_NDF_* = 0.03) digestibilities was greater in cows fed the high-NDF diets (Table 3).

**Table 3:** Effects of *brachytic2* (*br2*) mutation of corn and control corn silage at 2 dietary NDF contents on apparent total-tract digestibility of nutrients and greenhouse gas emissions

Variable	Low NDF		High NDF		SEM	*P*-value^1^		
	*br2*	Tall	*br2*	Tall		Hybrid	NDF	H × F
NDF digestibility (%)	41.8	37.5	46.2	41.6	0.71	<0.01	<0.01	0.82
Starch digestibility (%)	98.5	98.8	99.3	99.3	0.10	0.09	<0.01	0.22
CP digestibility (%)	70.3	68.6	72.8	69.8	0.80	<0.01	0.03	0.43
CO_2_ (g/d)	16,970	16,750	16,841	16,353	492	0.48	0.60	0.79
O_2_ (g/d)	11,471	11,423	11,316	11,277	274	0.87	0.59	0.98
CH_4_ (g/d)	444	478	527	513	25.2	0.70	0.03	0.34
H_2_ (g/d)	2.48	2.29	2.26	1.95	0.22	0.23	0.17	0.77
CH_4_/DMI (g/kg)	16.5	17.5	19.7	20.3	0.98	0.46	<0.01	0.73
CH_4_/ECM (g/kg)	9.76	10.6	11.5	11.5	0.51	0.40	0.02	0.42

1 Hybrid = effect of corn silage hybrid; NDF = effect of dietary NDF content; H × F = interaction of dietary NDF content and corn silage hybrid.

Emissions of CO_2_, O_2_, CH_4_, and H_2_ are presented in Table 3. No differences were observed between hybrids on any of the gases measured (all *P_Hybrid_* > 0.10). Production (*P_NDF_* = 0.03), yield (*P_NDF_* < 0.01), and intensity (*P_NDF_* = 0.02) of CH_4_ were greater in cows fed the high-NDF diets compared with cows fed the low-NDF diets.

We found that silage from a corn hybrid containing the brachytic mutation enabled cows in peak lactation to produce more ECM than those fed silage from a conventional tall hybrid, regardless of whether the total diet was high or low in forage NDF. The *br2* corn hybrids exhibit different lignin allocation into the plant that could be associated with greater NDF digestibility, as has been evaluated after 48 h of incubation (Bourdoncle et al., 2023). In a previous study, we found that cows in mid lactation fed silages from *br2* corn hybrids in diets with ∼29% NDF, 24% forNDF, and 26% starch (all %DM) produced greater yields of milk, protein, and lactose and a lower milk fat percentage compared with cows fed tall CS (Sarmikasoglou et al., 2025). In another study, multiparous Holstein cows early in lactation fed *br2* CS had lower DMI but produced more milk than cows fed a tall CS in diets with similar CP, NDF, starch, and sugar contents (Catellani et al., 2026).

Gut fill is considered the main limiting factor of intake in cows during peak lactation (Allen, 1996). We fed cows at 76 ± 31 DIM with diets that were contrasting in forNDF (17% vs. 24%) to determine if they could eat more when fed silage from *br2* corn compared with conventional tall corn. We found that cows fed the high-NDF diets had lower intake, as expected, indicating that gut fill was in fact limiting intake. The main effect of feeding silage from *br2* corn compared with tall corn was also significant, but the interaction of hybrid with NDF level was significant at *P* = 0.08; hybrid had little effect on intake for cows fed high starch low-NDF diets, but *br2*, compared with tall, silage enabled 1.8 kg greater intake for cows fed the high-NDF (56% CS, 24% forNDF) diets. Apparently, *br2* CS was less filling than the tall CS. For most other variables, the interaction of hybrid and NDF level was not significant, and production was increased by feeding more starch (less NDF) or by feeding NDF with greater digestibility. Feeding diets with more starch increased yields of ECM and protein, and feeding silage from *br2* corn instead of tall corn increased yields of milk and protein. As expected, based on the work of Oba and Allen (1999) and the review on partitioning of Piantoni and VandeHaar (2023), feeding more starch, which stimulates insulin, promoted both milk and body gain, but feeding more digestible fiber (*br2* vs. tall CS) partitioned extra nutrients mostly toward milk.

Diets high in starch increase intake; however, high starch and high intake usually decrease the total-tract digestibility of fiber (Putnam and Loosli, 1959; Valadares Filho et al., 2000; NASEM, 2021). Consistent with this, cows fed the low-NDF high-starch diets in this study had lower total-tract NDF digestibility.

We found that feeding low-NDF diets decreased CH_4_ production (g/d), yield (g/ kg DMI), and intensity (g/kg ECM), as expected (NASEM, 2021). However, hybrid had no effect on any of these measures of CH_4_ emissions. Feeding CS with lower lignin content and greater digestible fiber, such as *bmr* hybrids, has been reported to lower CH_4_ yields from dairy cattle in at least one study (Hassanat et al., 2017); however, NASEM (2021) predicts greater CH_4_ production when cows eat more and greater CH_4_ yield when digested NDF is increased. The lignin content of the *br2* hybrid used in this study was only slightly lower than that of the tall hybrid (6.30% vs. 7.30% DM), which might explain the lack of a difference in CH_4_ emissions. This is the first in vivo study investigating the effect of *br2* corn hybrids on enteric CH_4_ emission in dairy cattle; thus, further research is warranted.

Our findings demonstrate that feeding silage from a *br2* corn hybrid to cows in peak lactation increases fiber digestibility, intake, ECM yield, and milk protein yield compared with feeding silage from conventional tall stature corn. Moreover, no effects on methane emissions were observed between the 2 hybrids. Overall, based on their standability, DM yields per hectare, and effects on milk production, we conclude that short-stature hybrids have the potential to be valuable silage sources for enhancing dairy farm productivity and profitability.
